# Construction of *Escherichia coli* strains with chromosomally integrated expression cassettes for the synthesis of 2^′^-fucosyllactose

**DOI:** 10.1186/1475-2859-12-40

**Published:** 2013-05-01

**Authors:** Florian Baumgärtner, Lyudmila Seitz, Georg A Sprenger, Christoph Albermann

**Affiliations:** 1 Institute of Microbiology, Universität Stuttgart, Allmandring 31, Stuttgart, 70569, Germany

**Keywords:** GDP-L-fucose, 2^′^-fucosyllactose, Chromosomal integration, Human milk oligosaccharides

## Abstract

**Background:**

The trisaccharide 2^′^-fucosyllactose (2^′^-FL) is one of the most abundant oligosaccharides found in human milk. Due to its prebiotic and anti-infective properties, 2^′^-FL is discussed as nutritional additive for infant formula. Besides chemical synthesis and extraction from human milk, 2^′^-FL can be produced enzymatically *in vitro* and *in vivo*. The most promising approach for a large-scale formation of 2^′^-FL is the whole cell biosynthesis in *Escherichia coli* by intracellular synthesis of GDP-L-fucose and subsequent fucosylation of lactose with an appropriate α1,2-fucosyltransferase. Even though whole cell approaches have been demonstrated for the synthesis of 2^′^-FL, further improvements of the engineered *E. coli* host are required to increase product yields. Furthermore, an antibiotic-free method of whole cell synthesis of 2^′^-FL is desirable to simplify product purification and to avoid traces of antibiotics in a product with nutritional purpose.

**Results:**

Here we report the construction of the first selection marker-free *E. coli* strain that produces 2^′^-FL from lactose and glycerol. To construct this strain, recombinant genes of the *de novo* synthesis pathway for GDP-L-fucose as well as the gene for the *H. pylori* fucosyltransferase *futC* were integrated into the chromosome of *E. coli* JM109 by using the λ-Red recombineering technique. Strains carrying additional copies of the *futC* gene and/or the gene *fkp* (from *Bacteroides fragilis*) for an additional salvage pathway for GDP-L-fucose production were used and shown to further improve production of 2^′^-FL in shake flask experiments. An increase of the intracellular GDP-L-fucose concentration by expression of *fkp* gene as well as an additional copy of the *futC* gene lead to an enhanced formation of 2^′^-FL. Using an improved production strain, feasibility of large scale 2^′^-FL production was demonstrated in an antibiotic-free fed-batch fermentation (13 l) with a final 2^′^-FL concentration of 20.28 ± 0.83 g l^-1^ and a space-time-yield of 0.57 g l^-1^ h^-1^.

**Conclusions:**

By chromosomal integration of recombinant genes, altering the copy number of these genes and analysis of 2^′^-FL and intracellular GDP-L-fucose levels, we were able to construct and improve the first selection marker-free *E. coli* strain which is capable to produce 2^′^-FL without the use of expression plasmids. Analysis of intracellular GDP-L-fucose levels identified the *de novo* synthesis pathway of GDP-L-fucose as one bottleneck in 2^′^-FL production. In antibiotic-free fed-batch fermentation with an improved strain, scale-up of 2^′^-FL could be demonstrated.

## Background

With approximately 5 to 15 g l^-1^, human milk oligosaccharides (HMOs) are the third most abundant solid component of human milk after lactose and lipids [1]. All HMOs consist of a core structure containing at least lactose at the reducing end and can be extended with N-acetyllactosamine (LacNAc) units (type I or II) in a linear or branched form. The structural diversity of HMOs is further expanded by the extensive addition of L-fucose and/or sialic acid residues to terminal positions resulting in over 200 different compounds identified so far [2,3]. The concentrations of different HMOs and their total amount in human milk vary within the lactation phase and between individuals, which is believed to be partially based on genetic background [4,5]. Importantly, however, HMOs are not found in comparable abundances in other natural sources, like cow, sheep, or goat milk [6]. Several beneficial effects of HMOs on infants have been shown or suggested, including selective enhancement of bifidobacterial growth, anti-adhesive effects on pathogens and glycome-altering effects on intestinal epithelial cells [7-10]. Moreover, infants’ nutrition containing 2^′^-fucosyllactose (2^′^-FL) is associated with lower rates of diarrhea, making 2^′^-FL a potential nutritional supplement and therapeutic agent, if it were available in sufficient amounts and at a reasonable price [11].

Formerly, 2^′^-FL has been obtained via extraction from human milk or chemical synthesis [12,13], but the limited availability of human milk or the necessity of side group protection and deprotection in chemical synthesis, respectively, set limits to supply and cost efficiency. Thus, alternative sources of 2^′^-FL became of interest. With the identification and functional heterologous expression of an α1,2-fucosyltransferase (FutC) from *Helicobacter pylori*, which can utilize lactose as acceptor substrate [14], the research for a biotechnological alternative of 2^′^-FL production was enhanced.

FutC from *H. pylori* and the recently characterized WbsJ from *E. coli* O128:B12 are regio- and stereoselective α1,2-fucosyltransferases which connect the fucosyl-group in α-linkage to the 2-position of the galactosyl-residue of saccharides such as N-acetyllactosamine or lactose [15,16].

**Figure 1:**
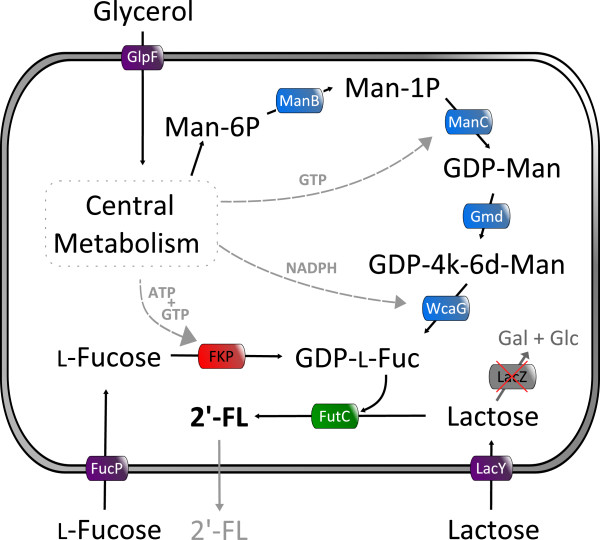
**Metabolic pathways for the whole cell biosynthesis of 2′-fucosyllactose (2′-FL) in *Escherichia coli*.** Salvage GDP-L-fucose pathway enzyme is depicted in red, *de novo* GDP-L-fucose synthesis pathway enzymes in blue and α1,2-fucosyltransferase enzyme in green. Enzymes and molecules are abbreviated as follows: Gal, D-galactose; GDP-4k-6d-Man, GDP-4-keto-6-deoxymannose; GDP-Man, GDP-α-D-mannose; GDP-L-Fuc, GDP-β-L-fucose; Glc, D-glucose; FucP, fucose permease; GlpF, glycerol MIP channel; LacY, lactose permease; Man-1P, α-D-mannose-1-phosphate; Man-6P, α-D-mannose-6-phosphate; ManB, phosphomannomutase; ManC, mannose-1-phosphate guanylyltransferase; Gmd, GDP-mannose 4,6-dehydratase; WcaG, GDP-fucose synthase; FutC, α1,2-fucosyltransferase; LacZ, β-galactosidase.

To obtain GDP-L-fucose for an *in vivo* synthesis of 2^′^-FL in *Escherichia coli*, the salvage pathway and/or the *de novo* synthesis pathway can be employed (Figure 1). The salvage pathway, catalyzed by the bifunctional enzyme Fkp with L-fucose kinase and L-fucose-1-phosphate guanylyltransferase activity, initially was believed to be present only in eukaryotes but today it also known from *Bacteroides fragilis*[17]. Fkp uses GTP, ATP, and L-fucose as substrates. Utility of the salvage pathway for GDP-L-fucose production has been demonstrated by complementation of *E. coli* cells with a disrupted GDP-L-fucose *de novo* synthesis pathway [18]. The *de novo* synthesis pathway, found in bacteria, mammals, and plants, transforms mannose-6-phosphate via mannose-1-phosphate, GDP-mannose, and GDP-4-keto-6-deoxymannose to GDP-L-fucose. Recombinant expression of the *de novo* pathway biosynthesis genes has been used for the *in vitro* and *in vivo* synthesis of GDP-L-fucose [19-22].

An enzymatic *in vitro* fucosylation of lactose to 2^′^-FL was accomplished by the use of recombinant α1,2-fucosyltransferase (FutC) from *H. pylori* and enzymatically prepared GDP-L-fucose [14]. But in particular the efforts and expenses for the preparation of GDP-L-fucose make this approach inapplicable for a large-scale production of 2^′^-FL. A large-scale biotransformation system for fucosylated sugars depending on a mixture of permeabilized *Corynebacterium ammoniagenes* and different *E. coli* cells was demonstrated by Koizumi *et al.*[23]. In this system, *C. ammoniagenes* was utilized for GTP regeneration, whereas different *E. coli* strains, expressing genes for GDP-L-fucose biosynthesis and fucosyltransferase, were used for the production of GDP-L-fucose and fucosylated oligosaccharides.

Whole cell biosynthesis of 2^′^-FL was carried out employing *E. coli* strains that provide endogenous GDP-L-fucose and overexpress α1,2-fucosyltransferase. To obtain sufficient amounts of GDP-L-fucose for cytoplasmic fucosylation reactions, different strategies were followed. It has been shown that GDP-L-fucose formation can be achieved by overexpression of plasmid-borne genes (*manB, manC, gmd, wcaG*) of the GDP-L-fucose *de novo* pathway [24,25]. In another approach, Dumon *et al.*[26] used plasmids to overexpress the gene for the positive regulator protein of the colanic acid biosynthesis, RcsA in an *E. coli* strain with inactivated *wcaJ* gene encoding the UDP-glucose lipid carrier transferase. This resulted in synthesis of GDP-L-fucose but avoided the formation of colanic acid.

So far, all approaches for the whole-cell synthesis of fucosylated oligosaccharides relied on the expression of plasmid-borne genes to obtain the necessary proteins. Expression plasmids are expedient for cloning and short-term expression of recombinant genes, in particular for the maximum overproduction of a given protein. For the *in vivo* synthesis of a natural product in a bacterial host, where multiple genes have to be expressed, the use of expression plasmids has several disadvantages. Plasmids tend to structural and segregational instability, that can lead to the loss or inactivation of the genes to be expressed [27]. To maintain expression plasmids in the cell, selection markers, like antibiotic resistance genes or plasmid addiction systems, are required. Such selection systems lead to the loss of viability of plasmid-free cells, but cannot avoid segregational instability. Moreover the metabolic burden of plasmid replication and the strong overexpression of certain genes can lead to reduced growth rates and increased demand of energy and metabolites for the additional pathway [28]. Most importantly, in processes aimed for human nutrition, the use of antibiotics is either legally forbidden or undesired for cost reasons. In addition, removal of antibiotics in downstream processing is elaborate and expensive.

To avoid these disadvantages, stable insertion of recombinant genes into the chromosome of the bacterial host may be desired. The chromosomal integration allows the maintenance of recombinant genes without the use of selection marker. For *Escherichia coli,* several methods have been developed for the insertion of expression cassettes into chromosome and its use for the synthesis of value products [29-33].

In the present study, we have constructed an *E. coli* strain carrying individual expression cassettes of genes on the chromosome to allow synthesis of GDP-L-fucose or 2^′^FL. Using this strain as platform, experiments were carried out to identify possible bottlenecks in GDP-L-fucose supply and fucosylation activity. After chromosomal integration of an additional copy of the fucosyltransferase gene, fed-batch fermentation was conducted to obtain higher yields of 2^′^-FL on a 13 liter scale in an antibiotic-free process.

## Results

### Construction of 2^′^-FL producing *Escherichia coli* strains

To realize the whole-cell synthesis of 2^′^-fucosyllactose, *E. coli* JM109 was chosen as parent strain. *E. coli* JM109 is a β-galactosidase-negative but lactose permease positive strain. This feature allows lactose uptake while its further metabolism is blocked. To allow the intracellular synthesis of GDP-L-fucose, genes of the GDP-L-fucose *de novo* pathway were cloned and integrated into the chromosome as individual expression cassettes under the control of an IPTG-inducible P_tac_-promoter. For this purpose the reading frames of *manB*, *manC*, and *gmd*-*wcaG* were cloned into the expression vector pJF119ΔN, respectively. The resulting plasmids (pJF-manB, pJF-manC, pJF-gmdwcaG (Table 1)) were each equipped with a FRT-flanked antibiotic resistance gene (*cat* or *kan*) downstream of the biosynthesis gene. From the gained plasmids the expression cassettes, including a P_tac_-promoter, a proper Shine-Dalgarno sequence followed by the individual biosynthesis gene, a FRT-cat-FRT or FRT-kan-FRT resistance marker, and a transcription terminator sequence (from *rrnB*) were each amplified by PCR. The PCR primers (Additional file 1: Table S1) were chosen in a way that each PCR product of the individual expression cassettes is flanked on both sides by a 50–60 bp DNA sequence which is homologous to the individual target sequence on the chromosome of *E. coli* JM109. The PCR products were inserted successively into different sugar degradation loci on the chromosome as described previously [32]. After each round of expression cassette integration, the antibiotic resistance gene was removed by the transient expression of a Flp recombinase [34], resulting in the strain *E. coli* JM109 gwBC (Table 1). To verify the synthesis of GDP-L-fucose by JM109 gwBC, the strain was equipped with a gene for the α1, 2-fucosyltransferase from *H. pylori* via the expression vector pJF-futC. Production of 2^′^-FL could be detected in the culture after IPTG induction and addition of lactose (data not shown). No 2^′^-FL was seen in control experiments using *E. coli* JM109 pJF-futC.

**Table 1:** Bacterial strains and plasmids used in this study

**Strains**	**Relevant genotype or sequences**	**Source or reference**
*E. coli* DH5α	F^-^, ϕ80d, *lacZ*ΔM15, *endA*1, *recA*1, *hsdR*17(r_K_^-^m_K_^-^), *supE*44, *thi*-1, *gyrA*96, *relA*1, Δ(*lacZYA-argF*)U169	laboratory strain
*E. coli* JM109	F′ *traD36 proA*^*+*^*B*^*+*^*lacI*^*q*^*Δ(lacZ)M15/Δ(lac-proAB) glnV44 e14*^*-*^*gyrA96 recA1 relA1 endA1 thi hsdR17*	laboratory strain
*E. coli* JM109 gwBC	*E. coli* JM109, *rbsDK::manC, melAB::manB, xylAB::gmdwcaG*	this study
*E. coli* JM109 gwBC-F1	*E. coli* JM109, *rbsDK::manC, melAB::manB, xylAB::gmdwcaG, malEG::futC*	this study
*E. coli* JM109 gwBC-F1-fkp	*E. coli* JM109, *rbsDK::manC, melAB::manB, xylAB::gmdwcaG, malEG::futC, fucIK ::fkp*	this study
*E. coli* JM109 gwBC-F2	*E. coli* JM109, *rbsDK::manC, melAB::manB, xylAB::gmdwcaG, malEG::futC, fucIK ::futC*	this study
*E. coli* JM109 gwBC-F2-fkp	*E. coli* JM109*, rbsDK::manC, melAB::manB, xylAB::gmdwcaG, malEG::futC, fucIK::futC, araBAD::fkp*	this study
**Plasmids**		
pKD46	P_araB_ γ β exo (red recombinase), Amp^R^	[35]
pCP20	FLP^+^, λ cI857^+^, λ p_R_ Rep^ts^, Amp^R^, Cm^R^	[34]
pMJC54	pET16b, *B. fragilis fkp* gene, Amp^R^	[17]
pCAW48	pET11a, *S. enterica manB* gene, Amp^R^	Albermann & Piepersberg (unpublished)
pCAW49	pET11a, *S. enterica manC* gene, Amp^R^	Albermann & Piepersberg (unpublished)
pCAW55	pJOE2775, *H. pylori futC* gene, Amp^R^	[14]
pCAS30-FRT-cat-FRT	pJF119ΔN, *P. ananatis crtE* gene, FRT-sites, Amp^R^, Cm^R^	[36]
pJF-crtY-FRT-kan-FRT	pJF119ΔN, *P. ananatis crtY* gene, FRT-sites, Amp^R^, Kan^R^	[32]
pQE31-FRT-cat-FRT	pQE31, FRT-sites, Amp^R^, Cm^R^	[37]
pJF119ΔN	cloning vector, RBS, P_tac_, Amp^R^	[38]
pJF-manB	pJF119ΔN, *S. enterica manB* gene, Amp^R^	this study
pJF-manC	pJF119ΔN, *S. enterica manC* gene, Amp^R^	this study
pJF-gmdwcaG	pJF119ΔN, *E. coli gmd, wcaG* gene, Amp^R^	this study
pJF-futC	pJF119ΔN, *H. pylori futC* gene, Amp^R^	this study
pJF-fkp	pJF119ΔN, *B. fragilis fkp* gene, Amp^R^	this study
pJF-manB-FRT-cat-FRT	pJF119ΔN, *S. enterica manB* gene, FRT-sites, Amp^R^, Cm^R^	this study
pJF-manC-FRT-kan-FRT	pJF119ΔN, *S. enterica manC* gene, FRT-sites, Amp^R^, Kan^R^	this study
pJF-gmdwcaG-FRT-cat-FRT	pJF119ΔN, *E. coli gmd, wcaG* gene, FRT-sites, Amp^R^, Cm^R^	this study
pJF-futC-FRT-cat-FRT	pJF119ΔN, *H. pylori futC* gene, FRT-sites, Amp^R^, Cm^R^	this study
pJF-fkp-FRT-cat-FRT	pJF119ΔN, *B. fragilis fkp* gene, FRT-sites, Amp^R^, Cm^R^	this study

**Figure 2:**
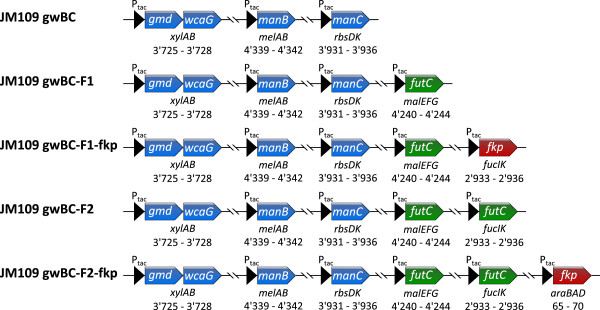
**Genetic modifications in *E. coli *strains compared in this study.** Chromosomally integrated genes in arrows, knocked-out sugar degradation genes and chromosomal position of knock-out by knock-in area in kb are given below corresponding integrated gene.

Based on strain JM109 gwBC, *futC* was integrated in the same way as described above, into the maltose locus *malEFG*, resulting in the strain JM109 gwBC-F1 (Figure 2). Shake flask cultivation of this strain, comprising the basic configuration of the required biosynthesis genes, revealed its ability to synthesize 2^′^-FL after IPTG induction and addition of lactose. In order to investigate rate limiting steps in the formation of 2^′^-FL by *E. coli* JM109 gwBC-F1 in whole cell biotransformation, concerning GDP-L-fucose supply or fucosylation reaction, the strain was further equipped with the GDP-L-fucose salvage pathway (*fkp*) and an additional copy of the fucosyltransferase gene (*futC*), resulting in the *E. coli* strains JM109 gwBC-F1-fkp, JM109 gwBC-F2, and JM109 gwBC-F2-fkp (Figure 2). By knocking-out the genes for fucose-isomerase (*fucI*) and fuculose-kinase (*fucK*) via insertion of *fkp* or the second copy *futC*, the strains lose the ability to degrade intracellular L-fucose.

### Determination of intracellular GDP-L-fucose levels and 2^′^-fucosyllactose formation

**Figure 3:**
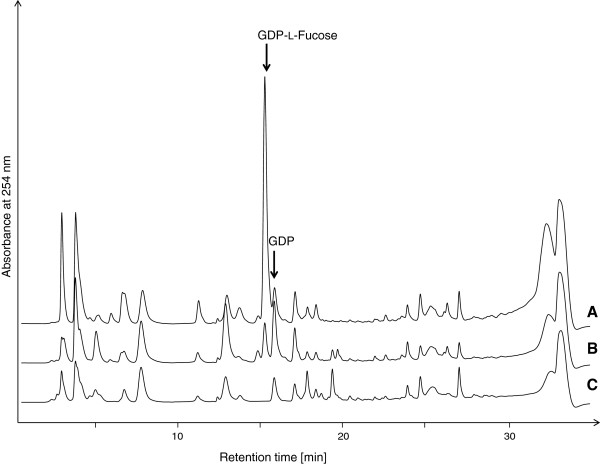
**HPLC analysis of intracellularly accumulated GDP-L-fucose. A)** Cell extract of *E. coli* JM109 gwBC-F1-fkp 12h after induction. **B)** Cell extract of *E. coli* JM109 gwBC-F1 12h after induction. **C**) Cell extract of *E. coli* JM109 12h after induction. Arrows indicate retention time of GDP-L-fucose and GDP, respectively.

**Figure 4:**
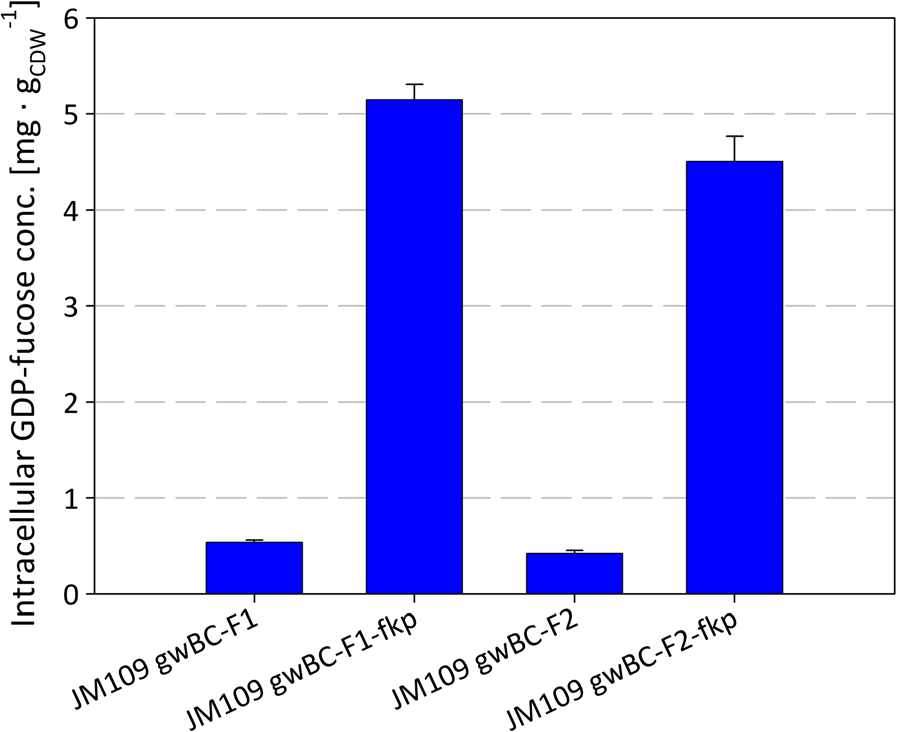
**Intracellular concentrations of GDP-L-fucose in *E. coli *strains with or without salvage synthesis pathway for GDP-L-fucose 12 hours after induction.**

To analyze the relation of GDP-L-fucose availability and 2^′^-FL production, intracellular GDP-L-fucose levels and cell dry weight concentrations (CDW) of the constructed 2^′^-FL producing strains were determined. The strains were cultivated in glycerol minimal medium, supplemented with 0.2% L-fucose. Whereas GDP-L-fucose was not detectable in the control strain JM109, intracellular accumulation was found in strains with the *de novo* pathway and about 10 times more GDP-L-fucose (about 5 mg g_CDW_^-1^) was found, when the *fkp* gene was present (Figures 3 and 4). GDP-L-fucose levels measured 4 hours after induction showed equal levels of GDP-L-fucose with a deviation of less than 5% to the values 12 hours after induction.

**Figure 5:**
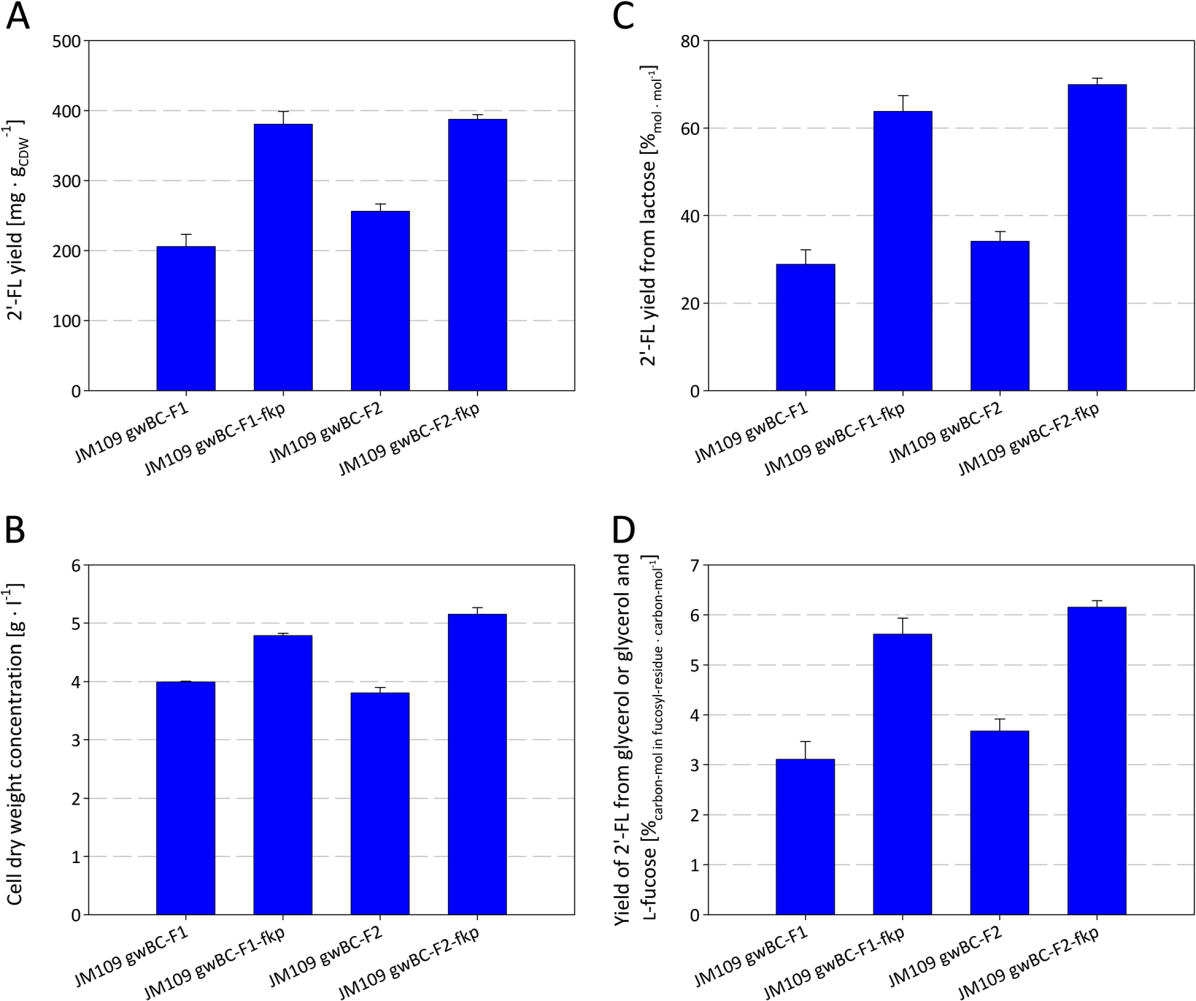
**Comparison of modified *E. coli *strains in shake flask experiments. A**) 2′-FL yield of batch cultivated strains with or without salvage GDP-L-fucose production 24 hours after induction. **B**) Cell dry weight concentrations corresponding to Figure 5A. **C**) Produced 2′-FL from lactose corresponding to Figure 5A. **D**) Percentage of carbon atoms converted from glycerol or glycerol and L-fucose to the fucosyl-residue of the produced 2′-FL corresponding to Figure 5A.

To determine the 2^′^-FL formation, shake flask cultivations were performed with glycerol minimal medium, supplemented with 0.2% lactose and, in strains carrying the *fkp* gene, 0.2% L-fucose. The experiments revealed that the strains that carry *fkp* show a significantly higher formation of 2^′^-FL and a significantly higher 2^′^-FL yield from lactose, than strains expressing the *de novo* pathway only (Figure 5A and C). The highest formation of 2^′^-FL was observed by *E. coli* JM109 gwBC-F2-fkp (387.76 ± 6.71 mg_2’-FL_ g_CDW_^-1^). Strain JM109 gwBC-F2, which relies on the *de novo* pathway only, produced about 30% less 2^′^-FL. That is, however, 15% higher than the production by the basic strain JM109 gwBC-F1 with only one *futC* copy on the chromosome. This shows that a higher intracellular GDP-L-fucose concentration as well as the additional *futC* expression cassette is beneficial to the increased formation of 2^′^-FL compared to the basic strain *E. coli* JM109 gwBC-F1. From cell dry weight determinations (after shake flask cultivations; Figure 5B) it became apparent that the biomass yield of strains that carry *fkp*, and thereby produce GDP-L-fucose mainly from L-fucose, is about 20% higher than strains that rely only on the *de novo* pathway for GDP-L-fucose formation. We take this for evidence that the subtraction of fructose-6-phosphate and NADPH by the *de novo* pathway reduces the carbon and energy pool of the cell and thus diminished the biomass yield. Strains with the additional salvage pathway, in contrast, use the fed L-fucose for GDP-L-fucose synthesis. Comparing ratios of carbon atoms found in glycerol or in glycerol and L-fucose with carbon atoms found in the fucosyl-residue of the produced 2^′^-FL, strains with the salvage synthesis pathway of GDP-L-fucose demonstrate significantly higher rates. The strain JM109 gwBC-F2-fkp shows 6.15 ± 0.13% of supplemented carbon atoms (without lactose) transferred to the fucosyl-residue of produced 2^′^-FL, while the strain JM109 gwBC-F2 without salvage synthesis pathway for GDP-L-fucose only shows 3.68 ± 0.24% (Figure 5D).

Genetic stability of the *E. coli* strain JM109 gwBC-F2 was analyzed under the assumption, that loss or mutation of the chromosomally integrated cassettes would result in shifts of the 2^′^-FL production rates or in the complete abolition of 2^′^-FL formation. For this, the strain JM109 gwBC-F2 was cultivated in minimal medium for several days with transfer to shake flask with new medium each 24 hours until it reached over 100 generations. When comparing 2^′^-FL yields in minimal medium with 0.2% lactose 24 hours after induction, cultures that had been cultivated for more than 100 generations still showed 95.01 ± 1.88% of the 2^′^-FL yield, observed in reference cultivations.

### Production of 2^′^-FL by fed-batch cultivation

**Figure 6:**
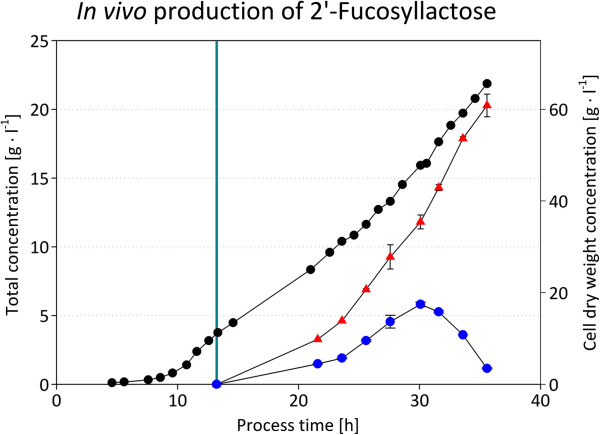
**2′-FL production in 10 liter scale glycerol-limited fed-batch fermentation of *E. coli *JM109 gwBC-F2.** Glycerol-, nitrogen- and lactose-feeds started with induction, marked by a turquoise vertical line. The glycerol and nutrient feed rates were adjusted to maintain a growth rate of μ = 0.1 h^-1^. Lactose feed rate was manually adjusted as described in the methods section. To allow a complete conversion of lactose into 2′-FL, the lactose feeding was stopped after a total process time of 30 hours. Symbols denote as follows: black circles, cell dry weight concentration; red triangles, concentration of 2′-fucosyllactose; blue circles, concentration of lactose.

In order to demonstrate a large scale synthesis of 2^′^-FL by a recombinant *E. coli* strain without expression plasmids and the use of any selection marker, the strain *E. coli* JM109 gwBC-F2 was used in a 13.5 liter fed-batch cultivation (Figure 6). Glycerol was used as carbon- and energy-source, whereas lactose was used as substrate for fucosylation only. After glycerol consumption in the batch phase the culture was induced with IPTG and lactose, glycerol, and nutrient feeding were started. After 35.5 hours, a total 2^′^-FL concentration of 20.28 ± 0.83 g l^-1^ was produced by a final cell dry weight concentration of 65.61 g l^-1^. This equals a 2^′^-FL yield of 309.16 ± 12.61 mg g_CDW_^-1^, being in the same order of magnitude as the yield measured in the shake flask experiments. The space-time-yield of the 2^′^-FL formation over the whole process was 0.57 g l^-1^ h^-1^. Analysis of the culture broth showed an almost equal distribution of 2^′^-FL in the culture broth and in the cell pellet with 10.22 ± 0.38 g l_culture_^-1^ and 10.18 ± 1.71 g l_culture_^-1^, respectively. The final glycerol concentration of the fed-batch culture was below the detection limit and the final acetate concentration was 0.262 ± 0.002 g l_culture_^-1^. To confirm the synthesis of 2^′^-FL by *E. coli* JM109 gwBC-F2, the oligosaccharide product was isolated from 50 ml culture by activated charcoal and size-exclusion chromatography. The isolated oligosaccharide (400 mg) was analyzed by MS and NMR (Additional file 2: Figure S1) and results were in agreement with previously reported assignments of 2^′^-FL [14].

## Discussion

Oligosaccharides found in human milk have been shown to be beneficial for the infants’ health. HMOs have been reported to serve as selective growth factor for specific bacteria such as Lactobacilli or Bifidobacteria [39] and they function as anti-adhesive antimicrobials that inhibit the adhesion of pathogenic viruses or bacteria to epithelial cells [40]. Furthermore, small quantities of HMOs may also be absorbed into the gastrointestinal tract where they modulate epithelial and immune cell response [41].

It was shown by *in vitro* and *in vivo* studies that 2^′^-FL has an anti-adhesive effect against *Campylobacter jejuni* and thereby lowers the risk of bacterial diarrhea caused by this organism [11,42]. In addition, 2^′^-FL selectively promotes the growth of bifidobacteria [43]. Due to these important biological activities of HMOs, efficient ways for HMO synthesis are needed. Currently, both chemical and enzymatic approaches are used for the formation of 2^′^-FL [13,14,25,44,45]. Especially the enzymatic formation of 2^′^-FL using a recombinant *in vivo* biosynthesis approach is to the best advantage, because co-factors like NADPH and GTP, needed for the enzymatic synthesis of GDP-L-fucose, can be regenerated in situ by the metabolism of the cell. For the *in vivo* production of 2^′^-FL by *E. coli* the expression of a GDP-L-fucose biosynthesis pathway and of a α1,2-fucosyltransferase are prerequisites. In previous studies, the intracellular formation of GDP-L-fucose in *E. coli* was achieved by the expression of the *de novo* biosynthesis genes *manB*, *manC*, *gmd*, and *wcaG* or by the RcsA-mediated activation of the native *de novo* pathway [24,26,46]. The α1,2-transfer of the L-fucosyl group from GDP-L-fucose to lactose can be achieved by the heterologous expression of the promiscuous α1,2-fucosyltransferase FutC from *H. pylori*[14,45,47].

So far, plasmids have been used for the expression of a heterologous 2^′^-FL biosynthesis pathway in *E. coli* strains, but the use of expression plasmids, in particular for large scale bioreactor cultivation, has several disadvantages. Plasmids tend to structural and segregational instability and therefore require a selection marker, like an antibiotic resistance gene, to maintain propagation of the plasmid in the cell [27]. For applications in food industry, especially the addition of antibiotics is undesirable. Furthermore the imbalanced expression of a few biosynthesis genes, either by high copy plasmids and/or strong promoters, can cause a metabolic burden effect and, thus, may lower product and/or biomass yield [28,48]. In the present study, to avoid the negative effects caused by plasmid-based expression, the required biosynthesis genes were integrated into the chromosome of *E. coli* as previously described [32]. Thus, up to six individual expression cassettes were established in the chromosome by replacing dispensable sugar degradation genes. Concerning the stability of the inserted biosynthesis genes we want to point out that chromosomal DNA is subject to evolutionary modifications by recombination or point mutation, but so far we have no evidence that the integrated expression cassettes tend to higher structural instability.

In contrast to previous works that used *E. coli* JM109 (DE3) as host for 2^′^-FL production [25,45], *E. coli* JM109, which has no detectable β-galactosidase activity, was employed to ensure the complete conversion of the lactose, assimilated via LacY, to 2^′^-FL (Figure 1). With the construction of the strain JM109 gwBC-F1, and the subsequent shake flask cultivations, we created an expression plasmid-free *E. coli* strain producing 206.16 mg g_CDW_^-1^ 2^′^-FL from lactose in minimal medium shake flask cultivation with glycerol as carbon source (Figure 5A). To improve 2^′^-FL productivity, strain development was used to identify possible bottlenecks in the *in vivo* fucosylation pathway. For the enzymatic formation of GDP-L-fucose the *de novo* synthetic and the salvage synthetic pathways have been described [17,19,20]. Here, in addition to the recombinant GDP-L-fucose *de novo* pathway, the salvage pathway was established in *E. coli* in order to modulate the intracellular GDP-L-fucose concentration. The salvage pathway requires the supply of L-fucose to the medium, from where the uptake is mediated by FucP. The intracellular degradation of L-fucose was prevented by the knock-out of *fucI* and *fucK* via the insertion of an expression cassette into *fucIK* (Figure 2). The additional GDP-L-fucose salvage pathway in a strain expressing the *de novo* pathway yielded an about 10 times higher intracellular concentration of the activated 6-deoxyhexose up to 5.1 mg g_CDW_^-1^. In a strain using only the de novo pathway, the lower GDP-L-fucose content maybe contributed to the feed-back inhibition of GDP-mannose-4,6-dehydratase by GDP-L-fucose [20,49,50]. Studies by Lee *et al.*[46,51] showed that the GDP-L-fucose content in the cell can be increased by the high expression of *manB*, *manC*, *gmd*, and *wcaG* as well as by the higher availability of NADPH in the cell with concentrations up to 2.8 mg g_CDW_^-1^.

Comparison of *E. coli* JM109 gwBC-F1 with *E. coli* JM109 gwBC-fkp-F1 showed an increase in 2^′^-FL productivity of over 80% with the salvage pathway of GDP-L-fucose synthesis indicating a bottleneck in the GDP-L-fucose supply for the fucosylation reaction in JM109 gwBC-F1 (Figure 5A). It should also be noted that the biomass yield of strains using the salvage pathway is significantly increased, reflecting a probable metabolite saving as the fucose-residue of 2^′^-FL mainly does not need to be withdrawn from anabolism, but is added with the fed fucose.

An increase in productivity of about 25% was observed with *E. coli* JM109 gwBC-F2 carrying an additional *futC* expression cassette on the chromosome compared to *E. coli* JM109 gwBC-F1. However, only a marginal increase in 2^′^-FL yield was seen by cultivations of *E. coli* JM109 gwBC-fkp-F2 compared to *E. coli* JM109 gwBC-fkp-F1. Thereby it can be concluded that, for the here reported basic 2^′^-FL producing strain *E. coli* JM109 gwBC-F1, both the expression of the fucosyltransferase as well as the supply of GDP-L-fucose represent rate limiting factors towards the *in vivo* synthesis of 2^′^-FL. Whereas the supply of GDP-L-fucose, that could be increased by the use of the salvage pathway, had the strongest effect on the *in vivo* 2^′^-FL formation.

In order to demonstrate an upscale of the 2^′^-FL synthesis using an expression plasmid-free production strain, a fed-batch cultivation of *E. coli* JM109 gwBC-F2 with glycerol as C- and energy source and lactose as substrate was conducted. Focusing on the production of high concentrations of 2^′^-FL combined with small amounts of remaining lactose to simplify the product purification process, a final product concentration of 20.28 ± 0.83 g l^-1^ could be achieved in a total process time of about 35.5 hours. With a final culture volume of 13.5 liters, a total amount of 273 g 2^′^-fucosyllactose and a space time yield of about 0.57 g l^-1^ h^-1^ were reached.

Compared to a fed-batch cultivation using *E. coli* with two expression plasmids described by Drouillard *et al.*[45], an increase in final concentration of almost 45% and an increase in productivity of about 83% were observed. This shows that an efficient *in vivo* fucosylation is possible even without the high expression of the recombinant 2^′^-FL biosynthesis genes. Our study emphasizes the need for balanced activities of biosynthesis enzymes for an efficient whole cell synthesis approach. A further improvement of the 2^′^-FL formation via the *de novo* pathway might be feasible by increasing the carbon flux towards GDP-L-fucose.

## Conclusions

In this study we constructed and improved an expression plasmid-free *E. coli* strain capable of the whole cell biosynthesis of the human milk oligosaccharide 2^′^-fucosyllactose from lactose and glycerol. The recombinant genes needed for the expression of a fucosyllactose biosynthesis pathway were integrated into the chromosome of *E. coli* JM109 as individual expression cassettes. By comparing strains expressing the GDP-L-fucose *de novo*- and the salvage-pathway, strains with one or two copies of the fucosyltransferase gene, and by measuring intracellular levels of GDP-L-fucose, we could identify bottlenecks in the 2^′^-FL biosynthesis for our production system. By a large-scale fed-batch cultivation we could demonstrate the efficient whole cell synthesis of 2^′^-fucosyllactose using a plasmid-free production strain without the use of antibiotics as selection markers.

## Methods

### Bacterial strains, media, and chemicals

All strains constructed are based on the *lacZ*-negative, but *lacY*-positive laboratory strain *E. coli* JM109 (Table 1 and Figure 2). If not stated otherwise, *E. coli* strains were grown at 37°C in LB medium. The minimal medium used for shake flask cultivation had the following composition: KH_2_PO_4_ 3 g l^-1^, K_2_HPO_4_ 12 g l^-1^, (NH_4_)_2_SO_4_ 5 g l^-1^_,_ MgSO_4_ · 7H_2_O 0.3 g l^-1^, CaCl_2_ · 2H_2_O 0.015 g l^-1^, NaCl 0.1 g l^-1^, glycerol 10 g l^-1^, FeSO_4_ · 7H_2_O/sodium citrate 15 ml l^-1^ (from the solution of 7.5 g l^-1^ FeSO_4_ · 7H_2_O and 100 g l^-1^ sodium citrate), thiamine 7.5 μg l^-1^, trace element solution 33 ml l^-1^[52]. Antibiotics for strain constructions were used at the following final concentrations: ampicillin 100 μg ml^-1^, kanamycin 50 μg ml^-1^, chloramphenicol 50 μg ml^-1^. Difco MacConkey agar base was purchased from Nordwald (Germany). 2^′^-fucosyllactose standard was purchased from IsoSep (Sweden); GDP-L-fucose was from Jennewein Biotechnologie (Germany). All other chemicals and reagents were from Sigma-Aldrich or Roth (Germany) and were of the highest purity available.

### Construction of plasmids

All used and constructed plasmids are listed in Table 1. The reading frames of *manB*, *manC*, *gmd*/*wcaG,* and *fkp* were individually cloned into the NdeI and BamHI sites of the expression vector pJF119ΔN [38], respectively. *manB* was subcloned from plasmid pCAW48 to yield pJF-manB. The gene *manC* was subcloned from plasmid pCAW49 to yield pJF-manC. A PCR product containing the reading frames of *gmd* and *wcaG*, as previously described [20], was digested with NdeI and BglII enzymes and ligated to vector pJF119ΔN DNA which had been digested with NdeI plus BamHI. This yielded plasmid pJF-gmdwcaG. The gene *fkp* was subcloned from plasmid pMJC54 to yield pJF-fkp. The reading frame of the fucosyltransferase gene *futC* was subcloned from plasmid pCAW55 into pJF119ΔN using NdeI and PstI restriction sites. The plasmid pJF-gmdwcaG was further treated with HindIII and ligated with a HindIII digested FRT-cat-FRT fragment derived from plasmid pCAS30-FRT-cat-FRT and transformed into *E. coli* DH5α. Plasmids pJF-manB and pJF-futC were treated with SphI and ligated with a SphI digested FRT-cat-FRT fragment derived from plasmid pCAS30-FRT-cat-FRT, respectively. Plasmid pJF-manC was hydrolyzed by SalI and ScaI and ligated with a 2.4 kb SalI/ScaI fragment received from plasmid pJF-crtY-FRT-kan-FRT containing FRT-kan-FRT cassette. Finally, a FRT-cat-FRT SalI fragment was subcloned from plasmid pQE31-FRT-cat-FRT into SalI hydrolyzed pJF-fkp.

### Chromosomal integration of expression cassettes

The individual expression cassettes of *manB*, *manC, gmd-wcaG, fkp* and *futC* including the FRT-flanked antibiotic-resistance genes from plasmid pJF-manB-FRT-cat-FRT, pJF-manC-FRT-kan-FRT, pJF-gmdwcaG-FRT-cat-FRT, pJF-fkp-FRT-cat-FRT, and pJF-futC-FRT-cat-FRT, respectively, were amplified by PCR. The oligonucleotide primers for the amplification of the expression cassettes are listed in Additional file 1: Table S1. For the chromosomal integration of the expression cassettes, the purified PCR products were consecutively transformed into *E. coli* JM109 carrying plasmid pKD46. Expression of the λ-Red enzymes and the preparation of competent cells were carried out as described previously [32,35]. Competent cells were electroporated with 0.2-0.4 μg of PCR product. After electroporation, the cells were resuspended in 1 ml LB-medium and incubated at 30°C for 12 h with shaking. Subsequently, the cell suspension was spread onto LB-agar plates containing chloramphenicol or kanamycin, according to the antibiotic resistance cassette in use. Antibiotic-resistant clones were assayed on MacConkey agar plates containing 1% of the respective sugar, corresponding to the targeted genes responsible for sugar degradation. Clones, that stayed pale on MacConkey agar after incubation over night at 37°C, were checked regarding correct recombination by control PCR. After each successful integration the antibiotic resistance cassette was eliminated using the plasmid pCP20 as previously described [34].

### Shake flask experiments for 2^′^-fucosyllactose production

To determine 2^′^-FL productivity, strains were cultivated in 250 ml shake flasks with 25 ml minimal medium containing 10 g l^-1^ glycerol as carbon source for growth. IPTG was added to a final concentration of 0.5 mM when cells had reached an optical density (OD_600_) between 0.4 and 0.6 and lactose (2 g l^-1^ final concentration) was added. Furthermore, cultures of strains expressing *fkp* were supplemented with L-fucose (2 g l^-1^ final concentration) at time of induction. 4 hours and 24 hours after induction, 10 ml culture samples were spun down (3500 rpm, 15 min); supernatants were stored at -20°C until HPLC analysis. Cell pellets were washed with 5 ml of deionized water and spun down as before. Afterwards, pellets were resuspended in 1 ml of deionized water and disrupted by boiling (5 min, 100°C). After another centrifugation (25 min at 5100 rpm), supernatants were stored at -20°C until HPLC analysis.

### Determination of 2^′^-fucosyllactose and lactose concentrations

Oligosaccharide concentrations in shake flask experiments and fed-batch fermentation were determined by HPLC of the 2-aminobenzoic acid-labeled saccharides. Samples and standards of 2^′^-FL and lactose were derivatized with anthranilic acid in presence of sodium cyanoborohydride [53]. HPLC analysis was performed on Dionex HPLC Instrument (Germany), installed with a Chromeleon Software, Gina autosampler, P580 pumps, and a RF2000 fluorescence detector. Products were analyzed on a Luna C18 (2) reversed phase column (250 mm × 4.6 mm, 5 μm, Phenomenex, Germany). Mobile phase consisted of solvent 1 (aqueous solution containing tetrahydrofuran 1% (v/v), orthophosphoric acid 0.425% (v/v) and 1-butylamine 0,3% (v/v)) and solvent 2 (acetonitrile). With a flow rate of 1 ml min^-1^, a gradient was applied with 97.5% solvent 1/2.5% solvent 2 at beginning and 80.5% solvent 1/19.5% solvent 2 after 25 minutes followed by a 4 minute isocratic period with 80.5% solvent 1/19.5% solvent 2 and a 5 minute equilibration period with starting conditions. Standards and samples were analyzed in duplicates and standard curves were created from 6 different concentrations.

### Measurement of intracellular GDP-L-fucose concentration

The *E. coli* strains JM109 gwBC-F1, JM109 gwBC-F1-fkp, JM109 gwBC-F2, and JM109 gwBC-F2-fkp were cultivated in shake flasks at 37°C in 100 ml minimal medium containing 1% glycerol. At an OD_600_ of 0.4 the gene expression was induced by addition of IPTG (0.5 mM final concentration). For cultures of *E. coli* JM109 gwBC-F1-fkp and *E. coli* JM109 gwBC-F2-fkp, L-fucose was added to a final concentration of 0.2% (w/v). 4 and 12 hours after induction, 25 ml of the culture were harvested by centrifugation (3500 rpm, 15 min), cells were washed with 5 ml deionized cold water, resuspended in 1 ml water and then disrupted by boiling (100°C, 2 min). After centrifugation (14000 rpm, 20 min), the supernatant was analyzed by HPLC. GDP-L-fucose was detected by UV-photometry at 254 nm (Dionex HPLC Instrument (as described above) installed with a diode array detector (Dionex, Germany)). As mobile phase a phosphate-buffer (solvent A: 30 mM potassium-phosphate, pH 6.0; 5 mM tetrabutylammonium hydrogen sulfate; 2% acetonitrile) and acetonitrile (solvent B) was used. As stationary phase, a reverse phase HPLC column (Gemini-NX C18, 250 × 4 mm (Phenomenex, Germany)) was used. The following flow gradient with a flow rate of 1.5 ml min^-1^ was applied: 0 to 10 min linear gradient from 100% solvent A/0% solvent B to 98% solvent A/2% solvent B, 10 to 30 min linear gradient from 98% solvent A/2% solvent B to 60% solvent A/40% solvent B, 30 to 30.10 min linear gradient from 60% solvent A/40% solvent B to 100% solvent A/0% solvent B, 30.10 to 35 min isocratic conditions to equilibrate the column at 100% solvent A/0% solvent B. GDP-L-fucose was identified by co-chromatography using authentic standard compound and by analysis of its UV–vis spectra. For the quantification of GDP-L-fucose the integrated peak areas were compared to HPLC standard curves of authentic standards.

### Assessment of genetic stability of *E. coli* JM109 gwBC-F2

Assuming, that loss or mutation of chromosomally integrated genes would lead to shifts or loss in productivity, genetic stability of the strain *E. coli* JM109 gwBC-F2 was analyzed by cultivating the strain for more than 100 generations before analyzing the 2^′^-FL yield. To cultivate the strain for over 100 generations, 10ml cultures of *E. coli* JM109 gwBC-F2 in glycerol minimal medium were incubated for 24 hours, before measuring OD_600_ and using the culture to inoculate a fresh shake flask to OD_600_ of 0.01. Passed generations were calculated by OD_600_. After 100 generations, the production of 2^′^-FL was determined as described above.

### Fed-batch cultivation

Scale-up of 2^′^-FL production employing *E. coli* JM109 gwBC-F2 was performed in a 30 l stirred-tank reactor (Bioengineering AG, Switzerland) with a batch volume of 8.4 l and a total feed volume of 5.1 l using mineral salt medium modified from Wilms *et al.*[54] and glycerol as carbon source. Medium used for preculture and batch fermentation contained (NH_4_)_2_SO_4_ 2.68 g l^-1^, (NH_4_)_2_-H-citrate 1 g l^-1^, glycerol 26.42 g l^-1^, K_2_HPO_4_ 14.6 g l^-1^, MgSO_4_ 0.241 g l^-1^, Na_2_SO_4_ 2 g l^-1^, NaH_2_PO_4_ · H_2_O 4 g l^-1^, NH_4_Cl 0.5 g l^-1^, thiamine 10 mg l^-1^, and trace element solution 3 ml l^-1^.

Glycerol-feed consisted of glycerol 629.08 g l^-1^, MgSO_4_ 23.5 g l^-1^, thiamine 0.6 g l^-1^ and trace element solution 119 ml l^-1^. As nitrogen-source, (NH_4_)_2_HPO_4_ 396 g l^-1^ was deployed and for 2^′^-FL production, a lactose solution 150 g l^-1^ was fed.

Trace element solution consisted of CaCl_2_ · 2 H_2_O 0.5 g l^-1^, FeCl_3_ · 6 H_2_O 16.7 g l^-1^, Na_2_-EDTA 20.1 g l^-1^, ZnSO_4_ · 7 H_2_O 0.18 g l^-1^, MnSO_4_ · H_2_O 0.1 g l^-1^, CuSO_4_ · 5 H_2_O 0.16 g l^-1^, and CoCl_2_ · 6 H_2_O 0.18 g l^-1^. Fed-batch cultivation was operated at 37°C at a pH regulated to 7.0 using 25% (v/v) ammonium hydroxide solution. The partial oxygen concentration (pO_2_) was maintained above 50% saturation by adjusting stirrer speed and aeration rate. The reactor pressure was maintained 500 hPa above surrounding pressure. 8 liters of batch medium were inoculated with 0.4 liters over-night preculture resulting in an OD_600_ of 0.22. After glycerol from batch medium had been consumed, indicated by a rise in dissolved oxygen, expression was induced by IPTG (0.5 mM final concentration) and feeding was started. Glycerol- and ammonia feeds were fed in a ratio of 81:19 with a carbon-limited growth rate (μset) of 0.1 h^-1^ according to equation 1:

(1) $$F\left(t\right)=\left[\left({µ}_{\mathrm{set}}/Y_{x/s}\right)+m\right]\cdot \left[\left(c_{x0}{\cdot V}_{0}\right)/c_{s0}\right]\cdot \exp\left({µ}_{\mathrm{set}}\cdot t\right)$$

with F [l h^-1^] being the feeding rate, μset [h^-1^] the desired growth rate, m [g g^-1^ h^-1^] the specific maintenance coefficient (calculated to be 0.04 g g^-1^ h^-1^), Y_X/S_ [g g^-1^] the specific yield coefficient of biomass from substrate (estimated from shake flask experiments to be 0.29 g g^-1^, data not shown), c_x0_ [g l^-1^] the biomass concentration at beginning of the feeding phase (7.9 g l^-1^), V_0_ [l] the working volume of the reactor at beginning of the feeding phase (8.4 l), c_s0_ [g l^-1^] the glycerol concentration in the feed (629.1 g l^-1^), and t [h] the feeding time [55]. Lactose feeding also began with induction and the flow rate was set to 40.5% of the feeding rate calculated for the glycerol feed for the first 10 hours after induction. It was set to 50.6% of the glycerol feeding rate calculated above for the next 6.5 hours and set to a fixed rate of 9.25 l h^-1^ for the final 6 hours of the process. These adjustments of the lactose feeding rate were chosen to prevent lactose limitation and extensive lactose accumulation. Lactose feeding was stopped short before glycerol feeding to minimize the amount of remaining lactose. Cell biomass was determined by measuring OD_600_ and cell dry weight.

### Determination of glycerol and acetate concentrations

Remaining glycerol and accumulated acetate were analyzed in the supernatant at the endpoint of fermentation. Glycerol was measured using a glycerol test-kit for foodstuffs and other materials (r-biopharm, Germany), whereas the acetate concentration was measured via HPLC as follows. Culture supernatant was boiled for 5 minutes and centrifuged for 15 minutes at 14000 rpm. After centrifugation, the supernatant was used for HPLC analysis on an Agilent 1100 Series HPLC system (Agilent Technologies, USA), equipped with ChemStation for LC 3D systems software, column heater, QuatPump, degasser, a RI-71 refractive index detector (Showa Denko, Japan), and an organic-acid column (300 mm × 8 mm, CS-Chromatographie Service, Germany). The mobile phase consisted of 5 mM sulfuric acid with a flow rate of 1 ml min^-1^. Samples were analyzed in duplicates and the standard curve was created from 5 different concentrations.

### Isolation and analysis of 2^′^-fucosyllactose

For further analysis, 2^′^-fucosyllactose was isolated from 50 ml of the fed-batch fermentation broth by activated charcoal chromatography and gel-filtration. For cell lysis, a 50 ml sample of fermentation broth was incubated at 100°C for 20 minutes. After subsequent centrifugation, supernatant was applied to a column (26 × 300 mm) filled with equal weight proportions of activated charcoal (Merck, Germany) and Celite 545 (Fluka, Germany). The column was washed with 2 column volumes of water and 2^′^-fucosyllactose was eluted by an ethanol gradient. Fractions containing 2^′^-fucosyllactose were pooled and further purified on a gel-filtration column (26 × 1000 mm) containing Bio-Gel P-2 (Bio-Rad, Germany). The purified 2^′^-fucosyllactose was analyzed by mass spectrometry and nuclear magnetic resonance spectroscopy. The mass was determined using a Bruker microTOF-Q system (Bruker, Germany) with electron spray ionization and the NMR spectra were recorded at 296 K using a Bruker Avance 500 spectrometer (Bruker, Germany).

## Competing interests

The authors declare that they have no competing interests.

## Authors’ contributions

FB, LS, and CA performed the experiments. CA initiated and coordinated the project. CA, FB, and GAS wrote the final manuscript. All authors approved the final version of the manuscript.

## Supplementary Material

Additional file 1: Table S1 Primer list. Click here for file

Additional file 2: Figure S1 ^1^H-NMR of isolated 2^′^-fucosyllactose. Click here for file
