# Supplementary material for: Construction of Escherichia coli strains with chromosomally integrated expression cassettes for the synthesis of 2′-fucosyllactose
Source: Microb Cell Fact. 2013 May 1;12:40. doi: 10.1186/1475-2859-12-40 (PMC3655002; doi:10.1186/1475-2859-12-40)
Supplement: Additional file 1: Table S1 — Primer list. [file 1475-2859-12-40-S1.pdf]

Table S1: Primer list

Primer	Sequence	PCR-template	PCR-product
malEFG-for	AAGGTAAACTGGTAATCTGGATTAACGGCGATAAAGGCTATAACGGTCTCGCTGTCAAGGCGCACTCCCGTTCTGG	pJF-futC-FRT-cat-FRT	P_{tac}-*futC*-FRT-cat-FRT for the replacement of *malEFG*
malEFG-rev	GATCGGTAATGCAGACATCACGGCAGCGGCGGCAAAGTCACCCCACAGGTAGTTTTCAGGGTTATTGTCTCATGAGCG		
fucIK-for	CCGAATAAAGTGAGGAATCTGTAATGAAAAAAATCAGCTTACCGAAAATTGGTCAAGGCGCACTCCCGTTCTGG	pJF-futC-FRT-cat-FRT or pJF-fkp-FRT-cat-FRT	P_{tac}-*futC*-FRT-cat-FRT or P_{tac}-*fkp*-FRT-cat-FRT for the replacement of *fucIK*
fucIK-rev	GTTAACCCTTCCAGCGCCGCGCGATAGAAATGCCCCCGCGTGGTATTAAGCAGGGTTATTGTCTCATGAGCG		
xylAB-for	GACGAACTGGTGTTGGGTAAGCGTATGGAAGAGCACTTGCGTTTTGCCGCCTGCTCAAGGCGCACTCCCGTTCTGG	pJF-gmdwcaG FRT-cat-FRT	P_{tac}-*gmdwcaG*-FRT-cat-FRT for the replacement of *xylAB*
xylAB-rev	ATTAAAGCTGGGACATTGCTCAGGCCGGTTAATTTCGCGGCCCAATCCAGACACCAGGGTTATTGTCTCATGAGCG		
rbsDK-for	ACCGTTCTTAATTCTGATATTTCATCGGTGATCTCCCGTCTGGGACATACCGATATGCATGCATCGATCACCACAATT	pJF-manC-FRT-kan-FRT	P_{tac}-*manC*-FRT-kan-FRT for the replacement of *rbsDACBK*
rbsDK-rev	ATTCACGCTAGCCCATACACCACGACTTCCTAAAGTAATCAGTACAGTACGGATACC CAGGGTTATTGTCTCATGAGCGGATAC		
melAB-for	CGGCGCATATTGCCCTGATGGACATTGACCCCACCCGCCTGGAAGAGTCGCATATTGTTCAAGGCGCACTCCCGTTCTGG	pJF-manB-FRT-cat-FRT	P_{tac}-*manB*-FRT-cat-FRT for the replacement of *melAB*
melAB-rev	AGCGCAACGATGGCTTTAAGTGTCAGATGGCTTCCTTCAGCAGACGGTTGATTGTCTGCAGGGTTATTGTCTCATGAGCG		
araBAD-for	TCTATAATCACGGCAGAAAAGTCCACATTGATTATTTGCACGGCGTCGTCGCTCAAGGCGCACTCCCGTTCTGG	pJF-fkp-FRT-cat-FRT	P_{tac}-*fkp*-FRT-cat-FRT for the replacement of *araBAD*
araBAD-rev	ATATAAGCGACCTCTTCCAGCACGATGGCGTTATGCACCGCATCTTCCGTCAGGATGGCCTTCTGCTTAATTTGATGCC		
