# Supplementary figures and images for: Construction of Escherichia coli strains with chromosomally integrated expression cassettes for the synthesis of 2′-fucosyllactose
Source: Microb Cell Fact. 2013 May 1;12:40. doi: 10.1186/1475-2859-12-40 (PMC3655002; doi:10.1186/1475-2859-12-40)

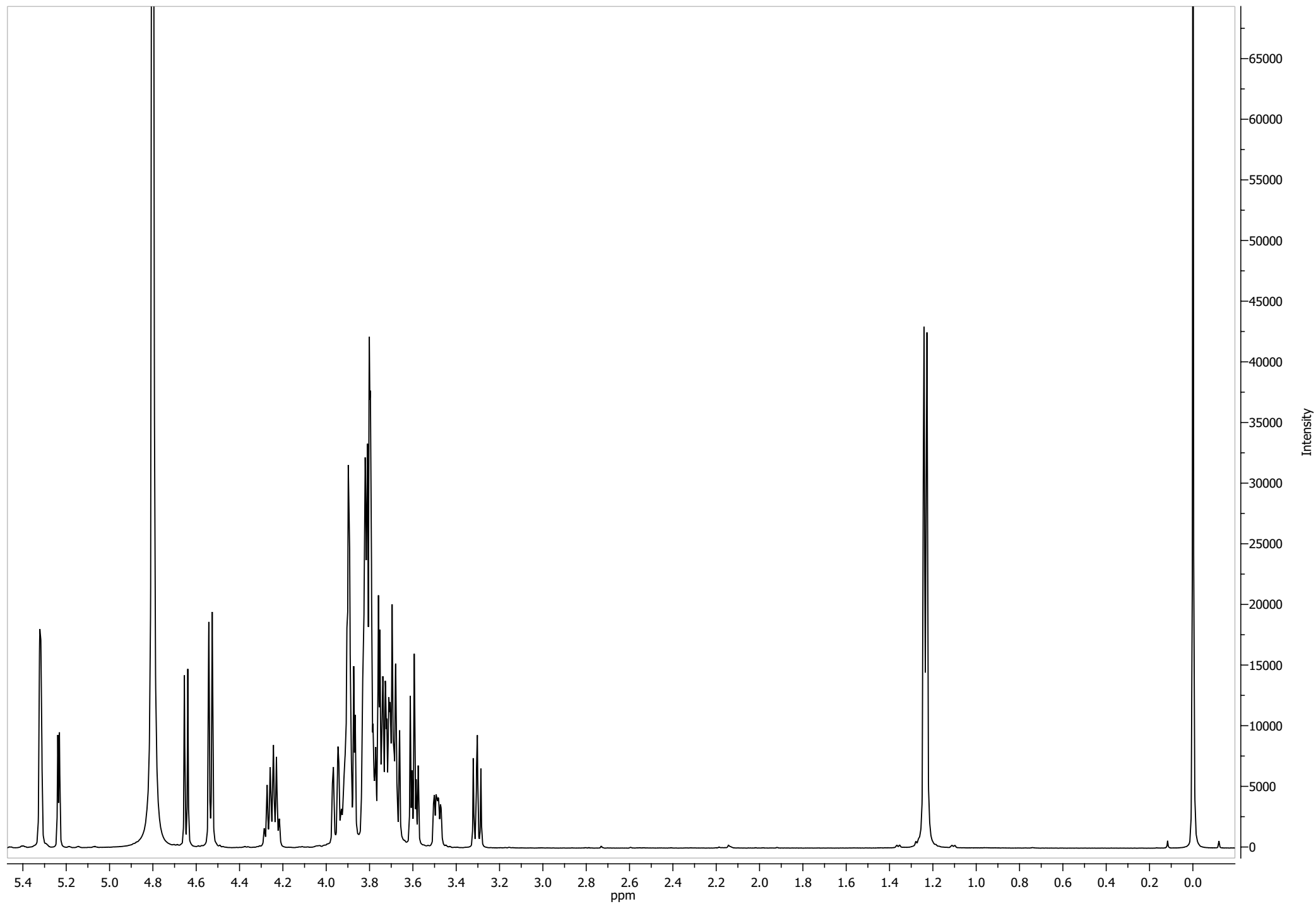

Supplement: Additional file 2: Figure S1 — 1H-NMR of isolated 2′-fucosyllactose. [file 1475-2859-12-40-S2.pdf]
